# *Grifola frondosa* may play an anti-obesity role by affecting intestinal microbiota to increase the production of short-chain fatty acids

**DOI:** 10.3389/fendo.2022.1105073

**Published:** 2023-01-17

**Authors:** Ruxiao Hu

**Affiliations:** Edible Fungus Institute of Hunan Province, Changsha, China

**Keywords:** *Grifola frondosa*, intestinal contents, microbial diversity, short-chain fatty acid, anti-obesity

## Abstract

**Background:**

*Grifola frondosa* (*G. frondosa*) is a fungus with good economic exploitation prospects of food and medicine homologation. This study aims to investigate the effects of *G. frondosa* powder suspension (GFPS) on the intestinal contents microbiota and the indexes related to oxidative stress and energy metabolism in mice, to provide new ideas for developing *G. frondosa* weight loss products.

**Methods:**

Twenty Kunming mice were randomly divided into control (CC), low-dose GFPS (CL), medium-dose GFPS (CM), and high-dose GFPS (CH) groups. The mice in CL, CM, and CH groups were intragastrically administered with 1.425 g/(kg·d), 2.85 g/(kg·d), and 5.735 g/(kg·d) GFPS, respectively. The mice in CC group were given the same dose of sterile water. After 8 weeks, liver and muscle related oxidative stress and energy metabolism indicators were detected, and the intestinal content microbiota of the mice was detected by 16S rRNA high-throughput sequencing.

**Results:**

After eight weeks of GFPS intervention, all mice lost weight. Compared with the CC group, lactate dehydrogenase (LDH) and malondialdehyde (MDA) contents in CL, CM, and CH groups were increased, while Succinate dehydrogenase (SDH) and Superoxide Dismutase (SOD) contents in the liver were decreased. The change trends of LDH and SDH in muscle were consistent with those in the liver. Among the above indexes, the change in CH is the most significant. The Chao1, ACE, Shannon, and Simpson index in CL, CM, and CH groups were increased. In the taxonomic composition, after the intervention with GFPS, the short-chain fatty acid (SCFA)-producing bacteria such as unclassified Muribaculaceae, *Alloprevotella*, and unclassified Lachnospiraceae increased. In linear discriminant analysis effect size (LEfSe) analysis, the characteristic bacteria in CC, CL, CM, and CH groups showed significant differences. In addition, some characteristic bacteria significantly correlated with related energy metabolism indicators.

**Conclusion:**

The preventive effect of *G. frondosa* on obesity is related to changing the structure of intestinal content microbiota and promoting the growth of SCFAs. While excessive intake of *G. frondosa* may not be conducive to the antioxidant capacity and energy metabolism.

## Introduction

*G. frondosa* [*Grifola frondosa* (Dicks.) Gray], also known as maitake, belongs to Basidiomycota, Hymenomycetes, Polyporales, Meruliaceae, and Ramalina. *G. frondosa* is a rare edible and medicinal fungus with effects of anti-obesity, anti-tumor, and regulating immune function, at the same time, it is rich in various bioactive components including polysaccharides, steroids and polyphenols (1). It has a long history of medicinal use in Oriental medicine in China, Japan, and India (2). In addition, *G. frondosa* has a delicious taste and is a good source of dietary fiber, protein, and carbohydrates (3).

The intestinal microbiota is a dynamic flora composed of 100 trillion microorganisms that inhabit the host’s intestinal tract (4). They play an irreplaceable role in various physiological activities, such as maintaining immune function, resisting colonization by pathogenic microorganisms, and assisting in nutrient absorption (5, 6). By secreting rich differential enzymes, some pharmaceutical components that do not have pharmacological activity can also be converted by the intestinal microbiota to form new active metabolites, which in turn have different biological effects on the body (7–9). More and more reports also indicated that intestinal microbiota might play a good intermediary role in the beneficial mechanism of *G. frondosa* (10). For example, Li et al. (11) reported that *G. frondosa* heteropolysaccharide could prevent non-alcoholic fatty liver disease by increasing the number of beneficial bacteria *Allobaculum*, *Bacteroides*, and *Bifidobacterium*. Chen et al. (12) reported that a new polysaccharide (GFP-N) extracted from *G. frondosa* could improve the intestinal microbiota of diabetic mice by increasing the abundance of *Akkermansia*, *Lactobacillus*, and *Turicibacter*. In addition, plant dietary fiber can be utilized and decomposed by the intestinal microbiota, partially absorbed by the microbiota itself, and partially converted into beneficial substances such as SCFAs (13). It has been reported that SCFAs can alleviate obesity, regulate intestinal pH, promote intestinal mucus production, and provide energy for epithelial cells (14–16). Among them, acetate, propionate, and butyrate are the intestines’ major SCFAs (14). Pan et al. (17) reported that the ethanol extract of *G. frondosa* can reduce the weight of rats fed with high-fat diet and increase the number of beneficial bacteria *Intestinimonas* and *Butyricimonas*, which are important producers of butyrate.

In daily life, people usually eat *G. frondosa* after simple decocting, or grind the *G. frondosa* into powder and use it as a flavoring agent (3). At the same time, the components of glycoprotein, ergosterol and pyrrolefronine in *G. frondosa* also have pharmacological effects of anti-obesity, anti-tumor or anti-diabetes (18–20). However, at present, most studies are on the polysaccharide components and their functions in *G. frondosa*, and there is little research on the effect of direct intervention of *G. frondosa* on intestinal microbiota. SOD and MDA are usually one of the important indicators to measure the body’s ability to remove oxygen free radicals and the level of oxidative damage (21). Some reports have shown that *G. frondosa* polysaccharides and polyphenols have the effect of anti-oxidative stress (22–24). LDH is a regulatory enzyme produced by glycolysis of sugars in the body in the absence of oxygen, which converts pyruvic acid into lactic acid, with reversibility (25, 26). SDH is a marker enzyme reflecting mitochondrial function, which can provide electrons for cell mitochondria and the aerobic and productive respiratory chain (27). In his master’s degree thesis, Li BG (28) reported the good potential role of *G. frondosa* fermentation broth in anti-fatigue and promoting energy cycle. Therefore, this study intervened the mice with different doses of GFPS to explore its effects on intestinal content microbiota, body weight, energy metabolism or oxidative stress-related indicators of liver and muscle in mice. Aiming to provide new ideas for the development of *G. frondosa* weight loss products and suggestions for people’s daily consumption.

## Material and methods

### Animals and feeding environment

In order to eliminate the gender influence (29), this study selected 20 SPF-grade male Kunming mice (20 ± 2 g), purchased from Hunan Slaccas Jingda Laboratory Animal Company (Hunan, China). The animals were raised at a temperature 23-25°C and humidity of 47-53% in the Experimental Animal Center of the Hunan University of Chinese Medicine.

### Medicine

*G. frondosa* is produced in Qingyun County, Lishui City, Zhejiang Province, and is the first fruiting mushroom product of *G. frondosa* stick cultivation (30). Take a certain amount of *G. frondosa*, dry it in an oven at 105-110°C to constant weight, grind it into powder, and pass it through a 60-mesh sieve to obtain *G. frondosa* powder. A proper amount of *G. frondosa* powder was heated with distilled water, and boiled for 5 min. Then, concentrated into low, medium and high dose GFPS of 0.053 g/ml, 0.106 g/ml and 0.215 g/ml, respectively. The solutions were cooled and stored in a refrigerator at 4 °C for standby.

### Animal grouping and feeding

**Figure 1:**
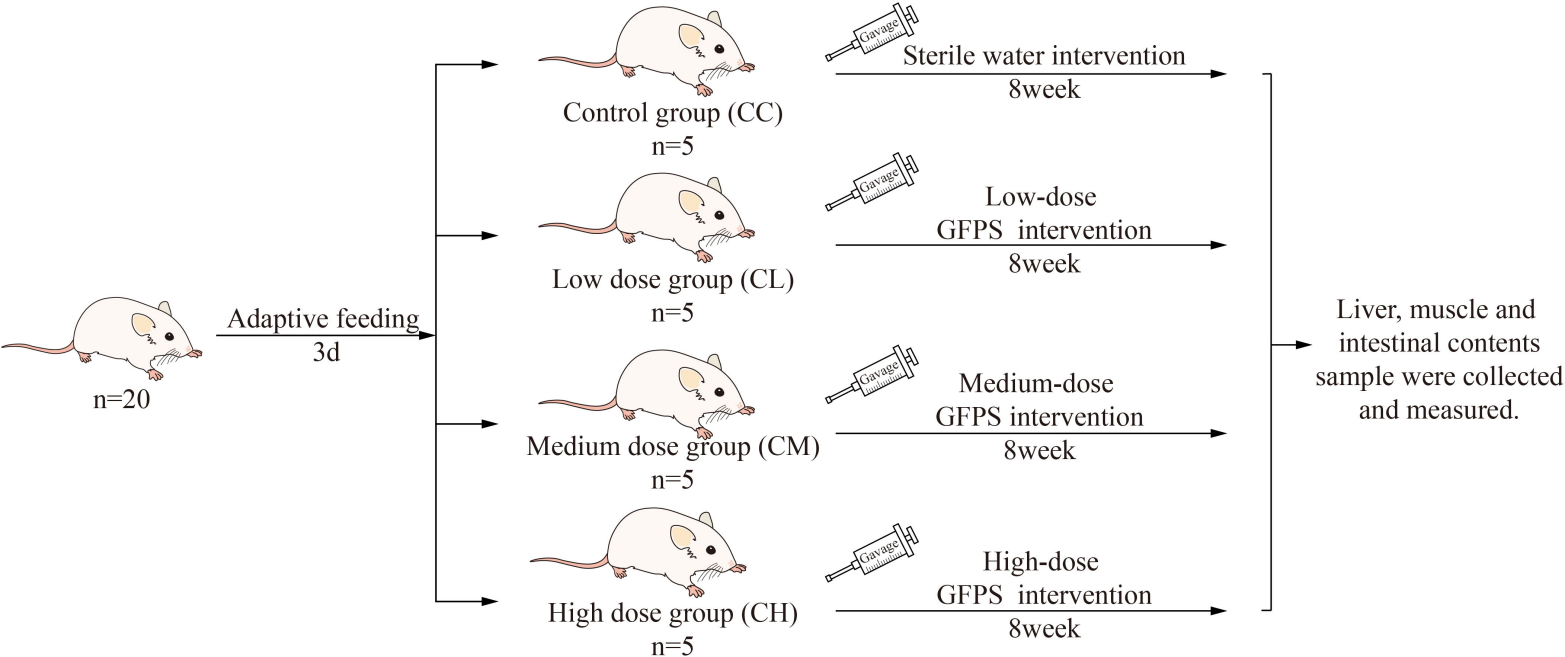
Experimental design and general conditions of the animals.

After 3 days of adaptive feeding, the mices were randomly divided into control group (CC), low-dose GFPS group (CL), medium-dose GFPS group (CM) and high-dose GFPS group (CH). The LD, MD and HD groups were given 0.4 mL low, medium and high dose GFPS by gavage, and CC Group was given the same frequency of sterile water by gavage, twice a day for 8 weeks. During this period, each mouse was weighed and recorded every week. The experimental procedures are shown in **Figure 1**. All animal experimental procedures were in the animal experimental protocol approved by the Institutional Animal Care and Use Committee of the Hunan University of Chinese Medicine.

### Biochemical indicators detection

At the end of the 8 weeks intervention, the mice were sacrificed on a sterile operating platform using cervical dislocation, and then the liver and muscles were taken out. According to the instructions of ELISA kits, the levels of SOD, MDA, LDH and SDH in the liver, and the levels of LDH and SDH in the muscle were detected using Rayto RT-6100 enzyme labeling analyzer. The kits were provided by Quanzhou kenuodi Biotechnology Co., LTD.

### Intestinal content sample collection

Under sterile conditions, the intestinal tissues from jejunum to ileum were longitudinally cut, and the intestinal contents were collected with forceps and stored at -80 °C for subsequent use (31).

### Extraction of total DNA, PCR amplification and high-throughput sequencing

The total microbial genomic DNA of the samples was extracted using a DNA extraction kit (MN NucleoSpin 96 So) through the steps of sample lysis, impurity removal by precipitation, inhibitor removal by filtration, DNA binding, membrane washing, drying, elution, etc. Using the extracted DNA as a template, the V3+V4 variable region of bacterial 16S rDNA was amplified with primers 338F (5’- ACTCCTACGGGAGGCAGCA-3’) and 806R (5’- GGACTACHVGGGTWTCTAAT-3’). The amplification reaction system consisted of 50 ng genomic DNA, 0.3 μL Vn F, 0.3 μL Vn R, 5 μL KOD FX Neo Buffer, 2 μL dNTP (2 mM each), and 0.2 μL KOD FX Neo, which were finally supplemented to 10 μl with ddH_2_O. Amplification conditions: denaturation at 95 °C for 5 min, rapid cooling to 50 °C, heating to 72 °C for 30 s, reacting for 40 s, then reacting at 72 °C for 7 min, and storing at 4 °C for 25 cycles. The amplified PCR products were purified, quantified, and homogenized. After the samples were mixed, they were subjected to column purification using OMEGA DNA purification column, and detected by 1.8% agarose gel electrophoresis. Use Monarch DNA glue recovery kit to cut glue and recover PCR products. The PCR products were sequenced by the Illumina Novaseq 6000 sequencing platform. All samples were processed by Beijing Biomarker Technologies Co, LTD.

### Bioinformatics

The obtained data were filtered by Trimmom (V0.33) (32), spliced by Usearch (v10.0) (33), and chimerism was removed by dada2 method (34) in QIIME2 (v2020.6) (35). Then effective sequences with similarity above 97% are clustered into an operational taxonomic unit (OTU), and the representative sequences of OTU are defined by classification. This study assessed the Alpha diversity of sample communities using ACE, Chao1, Simpson, and Shannon indices. The Beta diversity of sample communities was assessed using non-metric multidimensional scaling (NMDS) based on the unweighted unifrac distance. At the same time, the marked difference species in each group were screened by LEfSe, and the above visualization was completed with R v3.6.3.

### Correlation analysis

The correlation between the two variables can be expressed by the correlation coefficient. The closer the correlation coefficient is to 1, the greater the correlation between two elements, and the closer the correlation coefficient is to 0, indicating that the two elements are more independent. R version 3.6.3 is used to calculate Spearman rank correlation coefficient and draw heat map, network map, and scatter map.

### Statistical analysis

Experimental data were expressed as mean ± standard deviation. All the data were statistically analyzed by SPSS 21.0 statistical software. For comparison among multiple groups, the one-way analysis of variance was used to analyze the data that conformed to the normal distribution and homogeneity of variance, otherwise, the Kruskal-Wallis rank sum test was used. Pairwise comparison among multiple groups was performed using the LSD test. *p* < 0.05 indicated that the difference had statistical significance.

## Results

### Effects of GFPS on body weight

**Figure 2:**
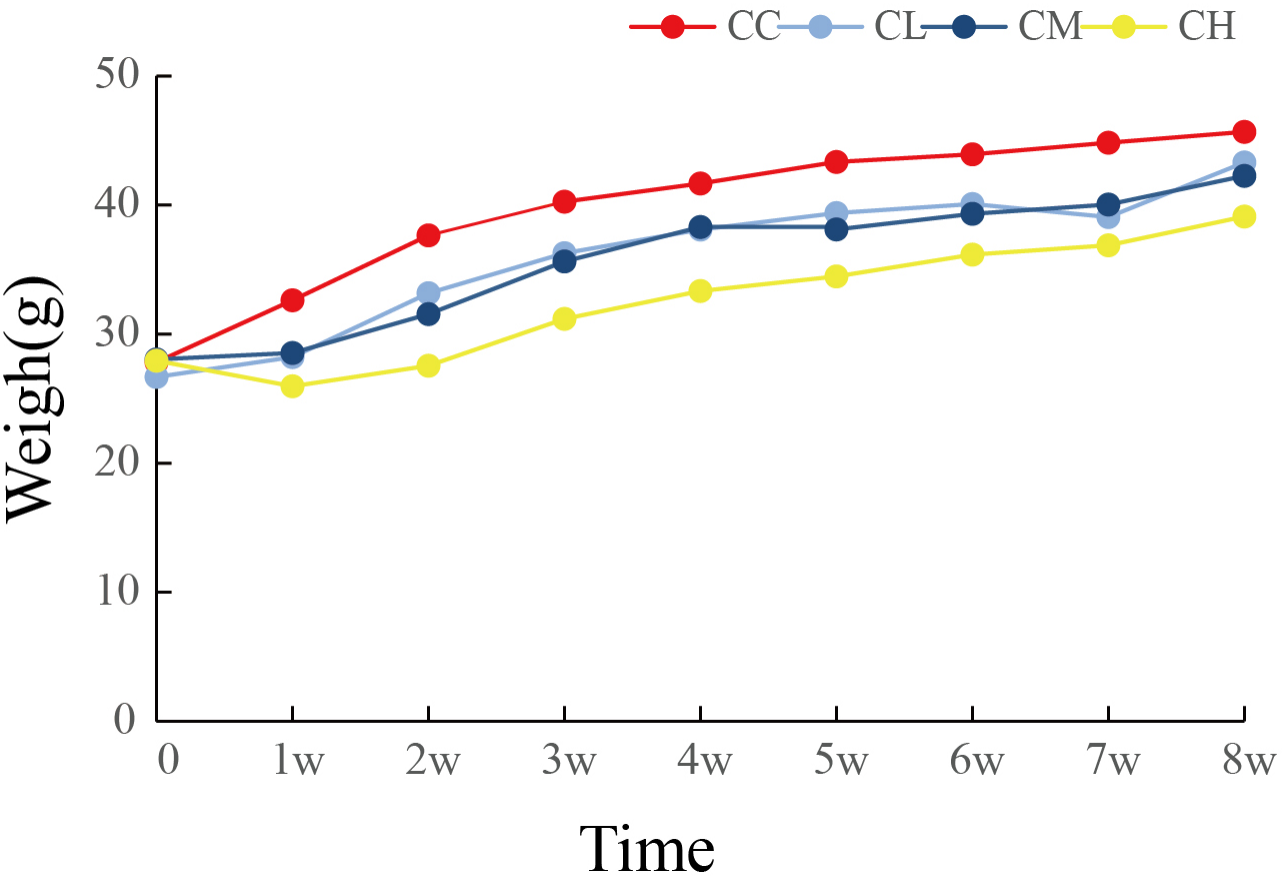
Weight changes of mice. CC: control group, CL: low-dose GFPS group, CM: medium-dose GFPS group, CH: high-dose GFPS group.

As shown in **Figure 2**, the body weight gain of mice after GFPS intervention was lower than that of CC group, and the CH and CM groups were significantly lower than CC group (*p*_CH_ < 0.01, *p*_CM_ < 0.05). Meanwhile, the trend of body weight gain in the CH group was the lowest among all GFPS intervention groups and significantly lower than that in the CL group (*p* < 0.05). This indicated that low, medium, and high doses of GFPS intervention had the effects of weight loss and lipid reduction, and the effect of high-dose of GFPS was the most significant.

### Effects of GFPS on LDH, SDH, SOD and MDA content in liver

**Figure 3:**
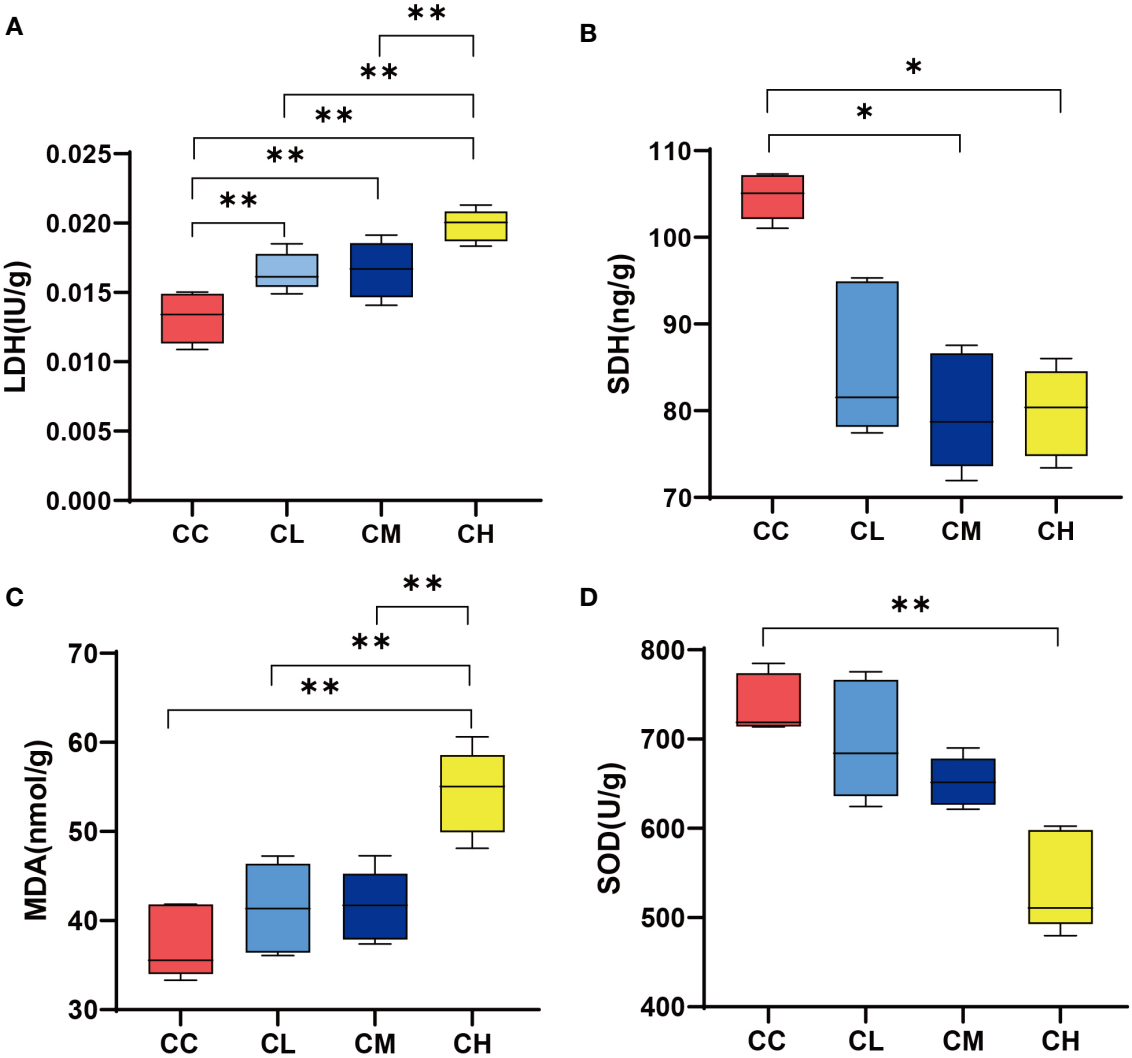
LDH, SDH, SOD, and MDA content in liver. **(A)** LDH content. **(B)** SDH content. **(C)** MDA content. **(D)** SOD content. CC: control group, CL: low-dose GFPS group, CM: medium-dose GFPS group, CH: high-dose GFPS group. (*p < 0.05, **p < 0.01).

LDH represented the anaerobic metabolism to some extent, while the up-regulation of SDH activity represented the acceleration of the tricarboxylic acid cycle and increase of Adenosine Triphosphate (25, 26). As shown in **Figure 3A**, LDH content in liver of different doses of GFPS intervention was significantly higher than that in CC group (*p* < 0.01). Meanwhile, LDH content in liver of CH group was significantly higher compared with the CL and CM groups. In terms of liver SDH content (**Figure 3B**), the SDH content after different doses of GFPS intervention was lower than that in the CC group, and the SDH content in the liver of the CM and CH groups was significantly lower than that in the CC group (*p* < 0.05). This indicated that the intervention of GFPS had an inhibitory effect on the energy metabolism level of liver cells in mice, and the intervention of high-dose of GFPS was the most effective. MDA is the final metabolite of lipid peroxidation, reflecting the body’s ability to be damaged by oxidation, while SOD indirectly reflects the body’s ability to remove oxygen free radicals (21). By comparing the levels of MDA and SOD activities, we could assess the effect of GFPS intervention on the antioxidant capacity of the liver in mice. As shown in **Figures 3C, D**, the MDA content in the CH group was significantly higher than that in other groups (*p*_CC_ < 0.01, *p*_CL_ < 0.01, *p*_CM_ < 0.01), and SOD content was significantly lower than that in the CC group (*p* < 0.01). It indicated that a high-dose of GFPS intervention might not have a beneficial effect on the antioxidant capacity of the liver in mice.

### Effects of GFPS on LDH and SDH content in muscle

**Figure 4:**
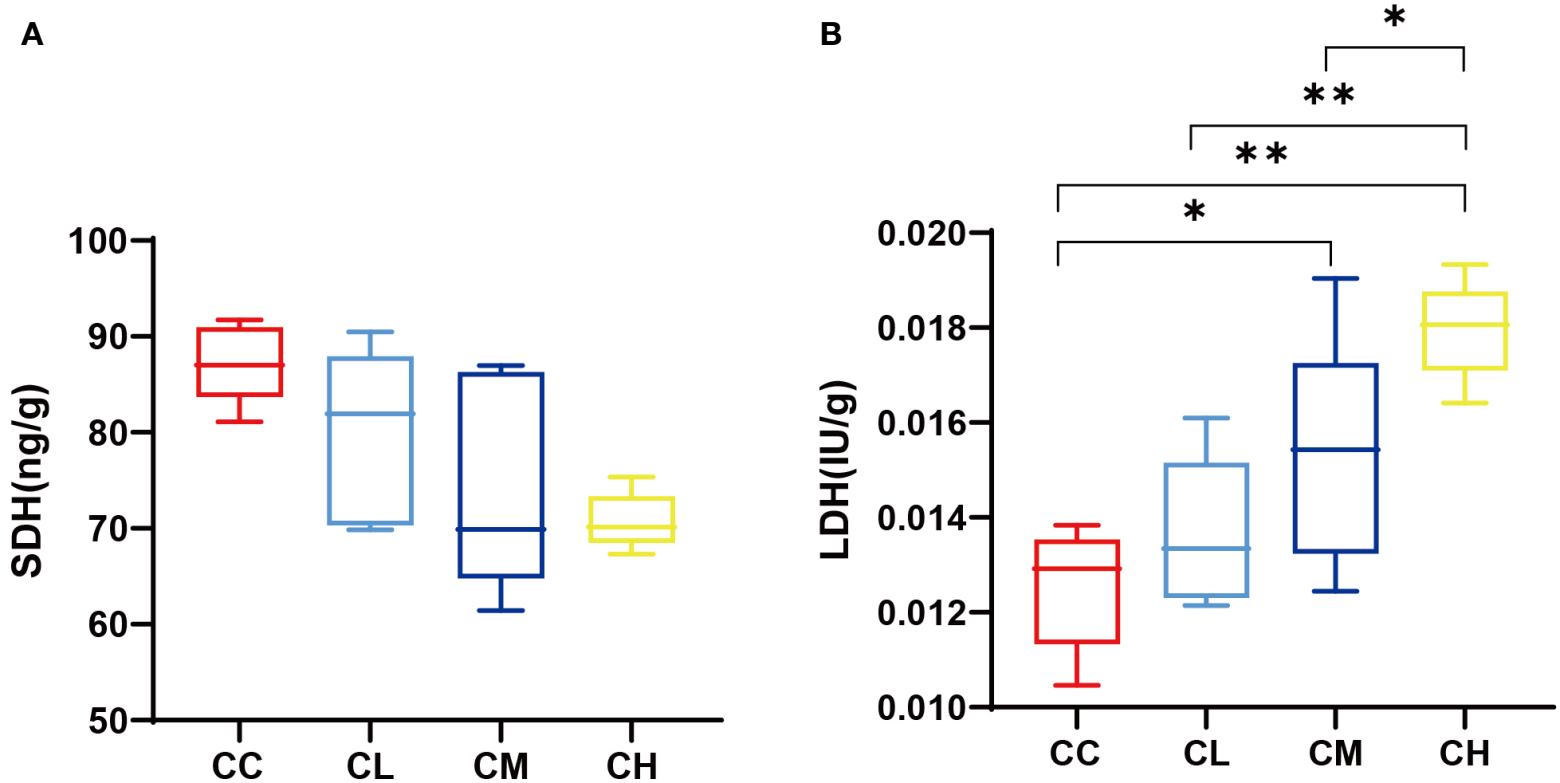
muscle SDH and LDH content. **(A)** SDH content. **(B)** LDH content. CC: control group, CL: low-dose GFPS group, CM: medium-dose GFPS group, CH: high-dose GFPS group. (*p < 0.05, **p < 0.01).

**Figure 4** shows that SDH content in the muscle of mice decreases with the increase of GFPS dose. At the same time, the LDH content in different mouse groups ranked CC < CL < CM < CH. Among them, the CC group had significant differences with CM and CH groups (*p*_CL_ < 0.05, *p*_CH_ < 0.01), and CH group had significant differences with CL and CM groups (*p*_CL_ < 0.01, *p*_CM_ < 0.05). This is consistent with the changing trend of LDH and SDH in mouse liver after different doses of GFPS. It indicated that the intervention of GFPS inhibited the energy metabolism of muscle cells in mice, and the inhibition was enhanced with the increase of dose.

### Effects of GFPS on intestinal content microbiota of mice

#### Effects of different concentrations of GFPS on the OTUs number of intestinal microbiota in mice

**Figure 5:**
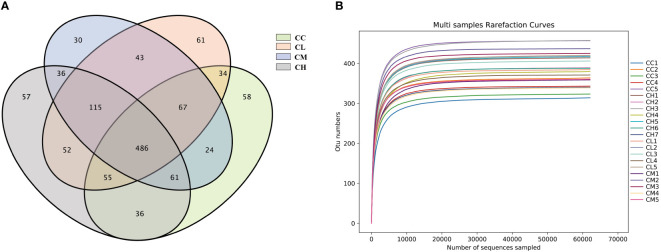
OTUs number and dilution curve of intestinal microbiota of mice. **(A)** OTUs number. **(B)** dilution curve. CC: control group, CL: low-dose GFPS group, CM: medium-dose GFPS group, CH: high-dose GFPS group.

As shown in **Figure 5A**, the numbers of OTUs obtained in CC, CL, CM, and CH groups were 821, 913, 862, and 898, respectively. The unique OTU numbers of CC, CL, CM and CH groups are 58, 61, 54 and 57 respectively. The dilution curve was used to assess whether sequencing was sufficient to cover all taxa and indirectly reflect the abundance of species in the sample. When the curve flattens out, it can be considered that the sequencing depth has covered almost all the species in the sample (36). As can be seen from **Figure 5B**, the dilution curve sequences of the four groups of samples tended to be gentle when the number was 10000. It shows that the amount of sequencing data is enough for the next analysis.

#### Effect of GFPS on the structure of intestinal microbiota in mice

In Alpha diversity analysis, Chao 1, ACE, Simpson, and Shannon are often used to evaluate richness and diversity (37). It can be seen from **Table 1** that the ACE, Chao 1, Simpson, and Shannon indexes of GFPS treated mice are slightly higher than CC group mice. The ACE and Chao 1 indexes of the CL group are higher than the CC, CM and CH groups, but there is no statistical significance. This result was consistent with the quantitative result of OTU, suggesting that the intervention of GFPS played a role in promoting the diversity and richness of intestinal content microbiota.

**Table 1:** Effect of GFPS on Alpha diversity index of intestinal content microbiota in mice.

Group	ACE	Chao 1	Simpson	Shannon
CC	361.078 ± 51.137	361.400 ± 51.247	0.976 ± 0.008	6.809 ± 0.411
CL	400.523 ± 19.347	400.700 ± 19245	0.981 ± 0.009	7.121 ± 0.214
CM	384.229 ± 39.146	384.200 ± 39.163	0.983 ± 0.004	7.103 ± 0.181
CH	382.732 ± 23.644	382.800 ± 23.760	0.981 ± 0.009	7.043 ± 0.336

All data are expressed as mean ± standard deviation. CC: control group, CL: low-dose GFPS group, CM: medium-dose GFPS group, CH: high-dose GFPS group.

**Figure 6:**
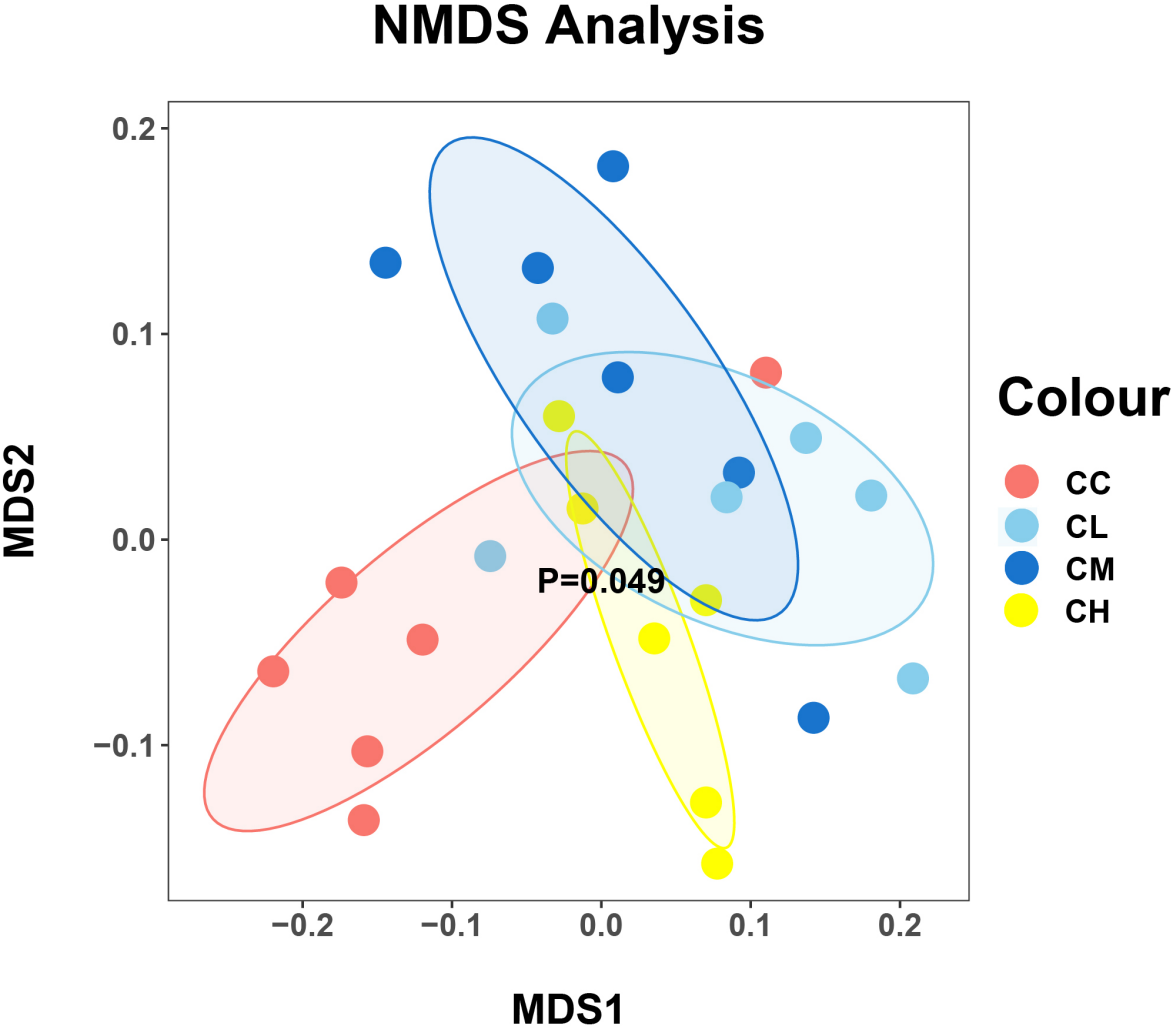
The beta diversity of mice intestinal mucosal bacteria. Each point represents a sample, and samples of different groups are represented by different colors. The closer the distance between two points is, the higher the similarity is between two samples, and the smaller the difference is. CC: control group, CL: low-dose GFPS group, CM: medium-dose GFPS group, CH: high-dose GFPS group.

In Beta diversity analysis, the nonlinear NMDS model can better reflect the nonlinear structure of ecological data (38). As shown in **Figure 6**, the distribution of samples in the CC and CH groups was relatively centralized, while that in CL and CM groups was relatively discrete. At the same time, the samples of the CC group are relatively separated from those of other groups, which indicated that the intervention of GFPS changed the community structure of the bacteria in the intestinal contents of mice.

#### Effect of GFPS on the structure and composition of intestinal contents microbiota in mice

**Figure 7:**
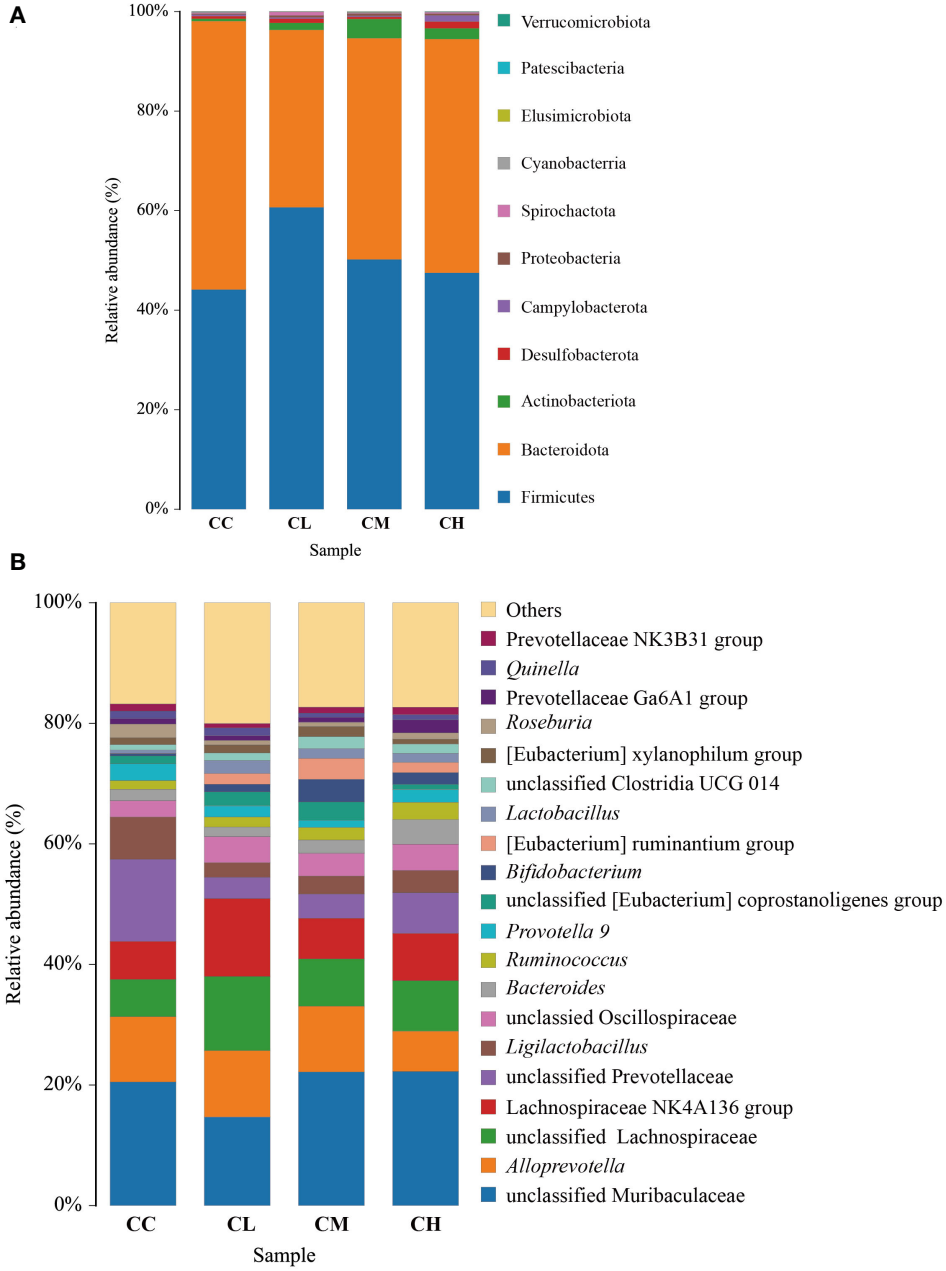
Relative abundance of bacteria in intestinal contents of mice after intervention with GFPS. **(A)** Level of phylum. **(B)** Level of genus. CC: control group, CL: low-dose GFPS group, CM: medium-dose GFPS group, CH: high-dose GFPS group.

At the phylum level (**Figure 7A**), the intestinal microbiota in the CC group consisted mainly of Bacteroidota (53.94%), Firmicutes (44.11%), Actinobacteriota (0.48%), Desulfobacterota (0.55%), and other low abundance proportion taxa. The abundance of Firmicutes (44.11% *vs* 51.99%, 60.68%, 50.17%) and Actinobacteriota (0.48% *vs* 2.23%, 1.41%, 3.86%) was higher in the CL, CM, and CH groups compared with the CC group. The abundance of Firmicutes (44.11% *vs* 51.99%, 60.68%, 50.17%) and Actinobacteriota (0.48% *vs* 2.23%, 1.41%, 3.86%) was higher in the CL, CM, and CH groups compared with the CC group. Meanwhile, the abundance of Bacteroidota (53.94% *vs* 42.73%, 35.60%, 44.45%) is lower.

At the genus level (**Figure 7B**), the dominant bacteria in CC, CL, CM, and CH groups were unclassified Muribaculaceae, accounting for 20.51%, 14.70%, 22.17%, and 22.27%, respectively. *Alloprevotella* was the second most common type, accounting for 10.84%, 11.00%, 10.92% and 6.68% respectively, while unclassified *Lachnospiraceae* accounted for 6.18%, 12.33%, 7.84% and 8.39% respectively.

#### Effect of GFPS on characteristic bacteria of intestinal contents in mice

**Figure 8:**
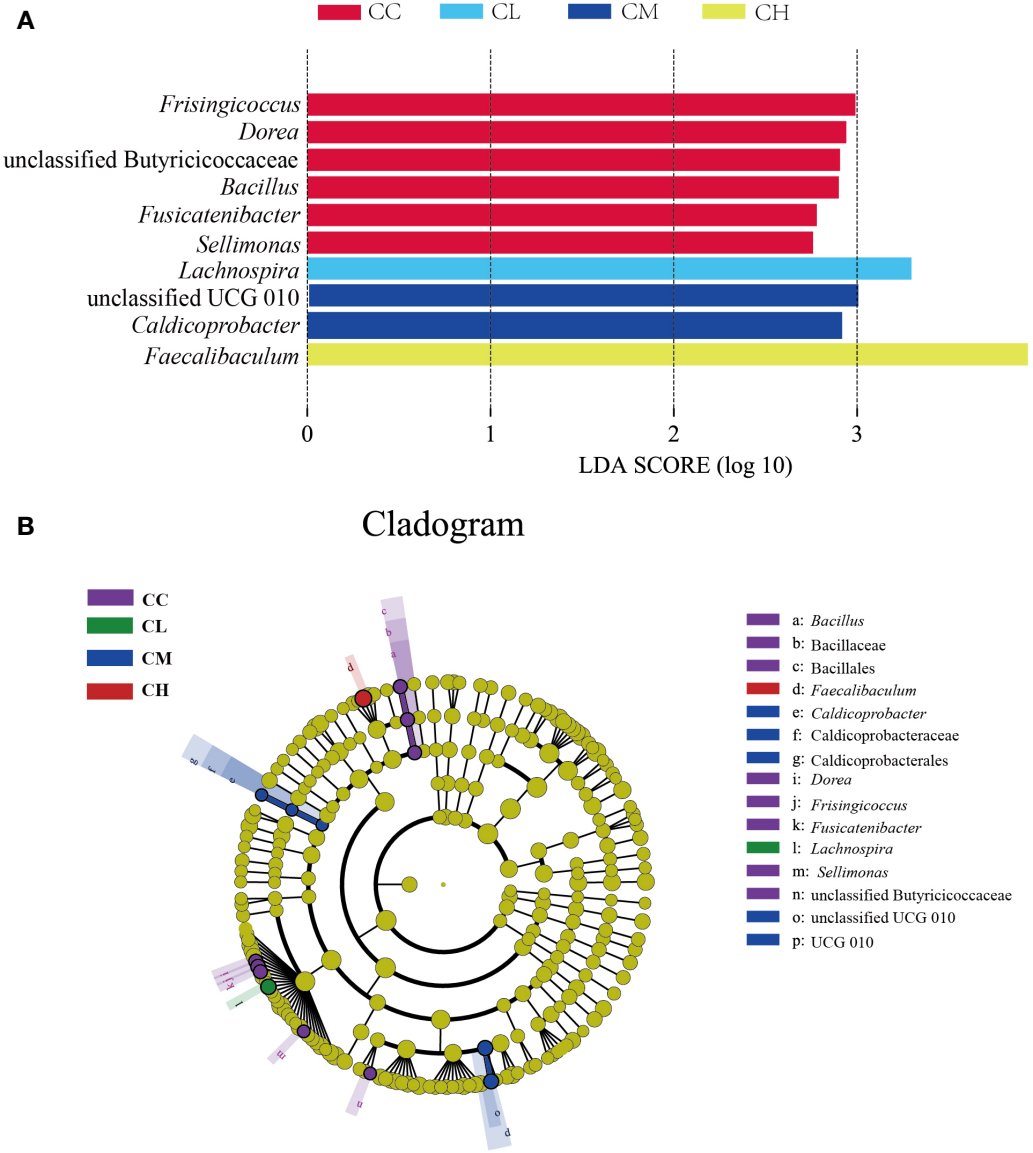
Characteristic bacteria of mice with GFPS intervention. **(A)** LDA score plots. **(B)** LEfSe analysis.

In order to further identify the characteristic microbiota of GFPS intervention, LEfSe analysis was performed on the community composition at each taxonomic level in different treatment groups. **Figure 8A** shows the characteristic bacteria when the logarithmic LDA threshold is 2, and the characteristic bacteria in CC group include *Frisingicoccus*, *Dorea*, unclassified Butyricicoccaceae, *Bacillus*, *Fusicatenibacter*, *Sellimonas*. The characteristic bacterium in the CL group was *Lachnospira*. The characteristic bacteria in the CM group were unclassified UCG 010 and *Caldicoprobacter*. The characteristic bacterium in the CH group was *Faecalibaculum*. The above results could explain that different doses of GFPS could change the intestinal content microbiota. In different classification systems (**Figure 8B**), the characteristic bacteria among the four groups showed significant differences.

### Correlation analysis of intestinal contents microbiota with liver and muscle index

**Figure 9:**
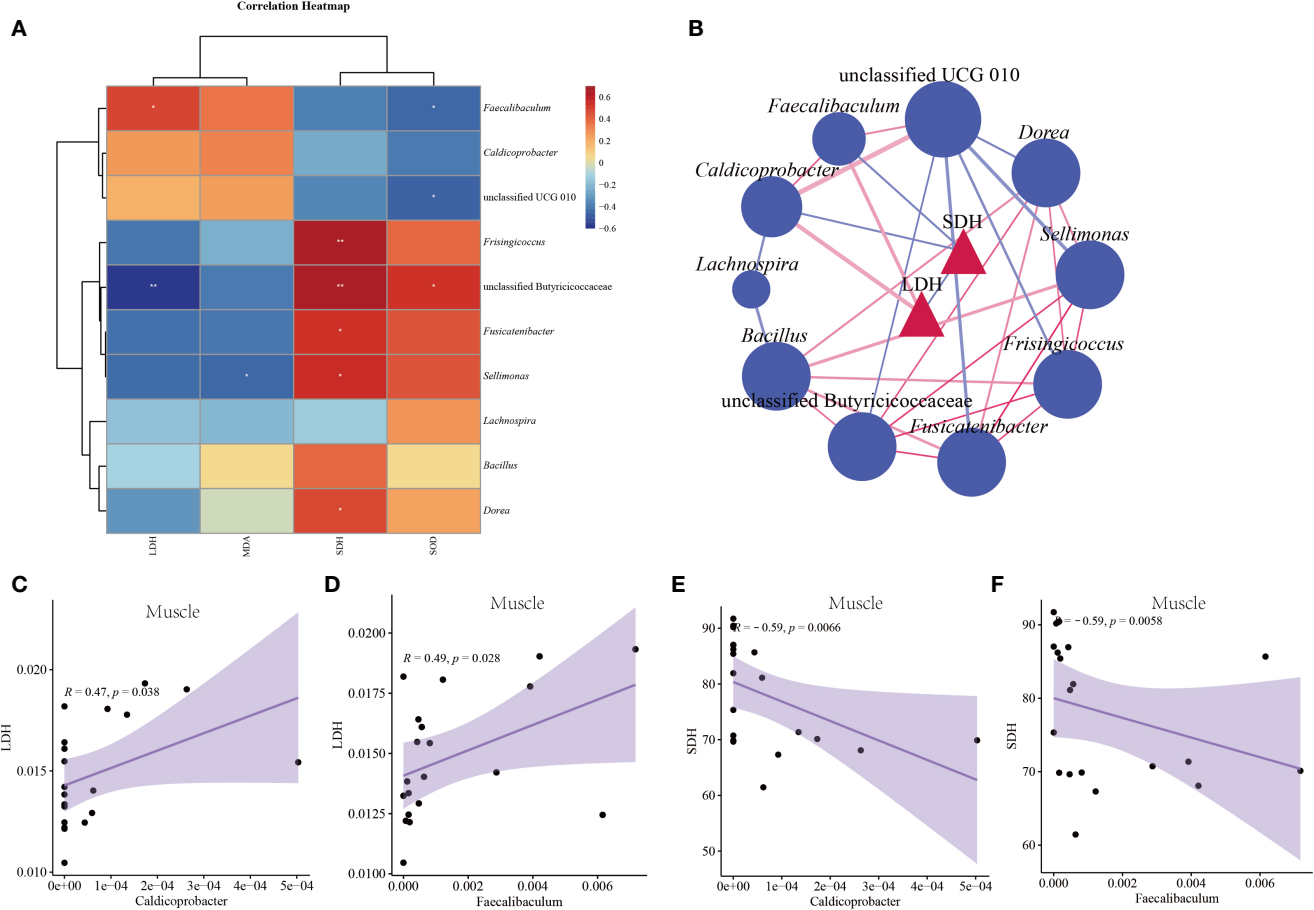
Correlation between characteristic bacteria of intestinal contents and indexes in mice. **(A)** Correlation heat map between characteristic bacteria and liver indexes. **(B)** Association network diagram of pathogenic bacteria and muscle indexes. **(C)** Scatter diagram of muscle LDH and *Caldicoprobacter*. **(D)** Scatter diagram of muscle LDH and *Faecalibaculum*. **(E)** Scatter diagram of muscle SDH and *Caldicoprobacter*. **(F)** Scatter diagram of muscle SHD and *Faecalibaculum*. CC: control group, CL: low-dose GFPS group, CM: medium-dose GFPS group, CH: high-dose GFPS group.

This study selected characteristic bacteria with logarithmic LDA threshold of 3 for correlation analysis between indicators. **Figure 9A** shows the correlation heat map between characteristic bacteria and liver indexes. LDH had a significant positive correlation with *Faecalibaculum* (*p* < 0.05) and a significant negative correlation with unclassified Butyricicoccaceae (*p* < 0.05). SDH was significantly positively correlated with *Frisingicoccus*, unclassified Butyricicoccaceae, *Fusicatenibacter*, *Sellimonas*, *Dorea* (*p* < 0.05). MDA was significantly negatively correlated with *Sellimonas* (*p* < 0.05). SOD had a significant positive correlation with unclassified Butyricicoccaceae (*p* < 0.05), and a significant negative correlation with *Faecalibaculum* and unclassified UCG 010 (*p* < 0.05). It could be seen from **Figures 9B–F** that LDH in muscle had a significant positive correlation with *Faecalibaculum* and *Caldicoprobacter* (*p* < 0.05). Muscle SDH significantly negatively correlated with *Caldicoprobacter* and *Faecalibaculum* (*p* < 0.05).

## Discussion

With the improvement of living standards and the strengthening of the concept of a healthy diet, people’s demand for healthy and functional foods is growing. Considering the side effects of some synthetic chemicals, multi-target and multi-channel natural plant products have broad application prospects in the development of functional foods and auxiliary drugs (39). Here, this study discussed the effects of GFPS intervention on oxidative stress, energy metabolism indicators, and intestinal microbiota in mice.

In the results of the liver oxidative stress index, the SOD contents decreased and MDA contents increased after the intervention with GFPS. Among them, the intervention of high-dose GFPS showed the most obvious change. This indicates that GFPS can inhibit the antioxidation of mouse liver. Notably, this differs from previous studies on the antioxidant effect of *G. frondosa*. For example, Men et al. (22) intervened the mice with acute liver injury with the polysaccharide extracted from the fruiting body of *G. frondosa*. They found that these *G. frondosa* polysaccharides not only had a protective effect on liver injury, but also decreased MDA content and increased SOD content in the liver. Another study (23) also found that *G. frondosa* polysaccharide intervention could significantly increase the liver antioxidant level of rats with hepatic fibrosis. We speculate that multiple complex components in *G. frondosa* may be the reason for this difference. It has been reported that *Faecalibaculum* has a negative correlation with SOD content, and can be used as a marker bacterium for intestinal oxidative stress (40). Oxidative stress improves cell permeability and causes LDH efflux from cells, thus leading to increased LDH activity (41). In experimental results, this was confirmed by increased LDH content in muscle and liver, which is also consistent with the significant positive correlation between *Faecalibaculum* and LDH in correlation analysis. and the contents of these enzymes in liver and muscle tissues have the LDH and SDH are representative enzymes of anaerobic respiration and aerobic respiration, respectively (42), same trend. This means that GFPS intervention reduced the energy metabolism of muscle and liver in mice to varying degrees, promoted anaerobic respiration, and had a certain dose dependence.

The diversity of intestinal microbiota is generally positively correlated with the quality of diet (43). The Alpha index and OTU results in this experiment showed that GFPS intervention promoted the diversity and richness of the intestinal content microbiota in mice. Meanwhile, the Beta analysis also showed that GFPS intervention changed the structure of intestinal microbiota. By further comparing the changes in the bacterial abundance of the intestinal contents among the four experimental groups, we could understand how GFPS affects the intestinal microbial environment. As the two largest taxonomic phylum in the intestinal microbiota, the increase in the ratio of Firmicutes to Bacteroidetes (F/B) is generally considered to be related to obesity (44). Two recent reports have shown that either *G. frondosa* polysaccharide or direct feeding *G. frondosa* can regulate lipid metabolism, reduce body weight, and is related to the TLR4/NF-κB signaling pathway (45, 46). In this research, the weight growth trend of mice after low, middle, and high doses of GFPS intervention decreased, which also proved this point. But interestingly, compared with the CC group, the F/B value of mouse microbiota in CL, CM, and CH groups increased.

From the perspective of taxonomic composition, the weight loss and intestinal health improvement effects of *G. frondosa* may be related to the promotion of the growth of SCFAs-producing bacteria, which can convert dietary fibers not absorbed by the body into metabolites SCFAs (47). SCFAs have been proved to stimulate energy consumption by promoting lipid oxidation, and the increase in SCFAs production can stimulate a large number of hormones and neural signals in different organs and tissue sites, thereby cumulatively inhibiting short-term appetite and energy intake (48, 49). In addition, SCFAs can also prevent diet-induced obesity by inhibiting the activity of Histone Deacetylase 3 in intestinal epithelial cells (15). Studies have shown that unclassified Muribacullaceae (50), unclassified lachnospiraceae (51), and *Alloprevotella* (52) are all intestinal SCFAs-producing bacteria. At the same time, compared to the CC group, the abundance of unclassified Muribaculaceae was increased in the CM and CH groups, and that of *Alloprevotella* was increased in the CL and CM groups. The abundance of unclassified Lachnospiraceae was increased after GFPS intervention. On the contrary, after high-dose intervention with GFPS, the abundance of *Alloprevotella* was decreased, but the characteristic bacterium *Faecalibaculum* in the CH group was also the producer of SCFAs (53). Studies have shown that *Faecalibaculum* has the effect of inhibiting the development of metabolic diseases (54). Combined with the results of oxidative stress, the MDA content of the CH group was significantly increased (*p* < 0.01), while the increased MDA content might damage the intestinal barrier (55), reduce the absorption of nutrients, and thus reduce body weight. This was also demonstrated by the lowest body weight in the CH group among the four groups (**Figure 2**). Furthermore, *Lachnospira* was a characteristic bacterium in the CL group, while *Lachnospira* could produce lactic acid and acetate, and lactic acid could become a metabolic substrate for bacteria to produce butyrate or propionate (56). The characteristic bacteria in LM group are *Caldicoprobacter* and unclassified UCG 010. *Caldicoprobacter* is a kind of bacteria that can degrade complex carbohydrates and ferment hemicellulose into lactic acid, ethanol, and hydrogen. Meanwhile, these products can be transformed into butyrate for colon cells to supply energy (57, 58).

In addition, there were some limitations in this study, such as the small sample size and the emphasis on the integrity and naturalness of *G. frondosa* in the experiment, and no specific discussion on the role of a component in *G. frondosa.* The intestinal microbiota is closely related to our health. In the future, metagenomic functional gene analysis will be used to further explore the relationship between *G. frondosa* and intestinal microbiota.

## Conclusion

In summary, *G. frondosa* can promote health and prevent obesity by changing the structure of intestinal content microbiota, promoting microbiota diversity and richness, and increasing the beneficial bacteria producing SCFAs. However, excessive intake of *G. frondosa* may promote oxidative stress response in mice and inhibit energy metabolism in muscle and liver tissue. Therefore, low-dose (1.425 g/kg·d) of *G. frondosa* may be a good choice for further experiment or product development.

## Data availability statement

The datasets presented in this study can be found in online repositories. The names of the repository/repositories and accession number(s) can be found below: https://www.ncbi.nlm.nih.gov/, PRJNA903652.

## Ethics statement

The animal study was reviewed and approved by the Institutional Animal Care and Use Committee of the Hunan University of Chinese Medicine.

## Author contributions

Experimental design, animal operation, data analysis, and paper writing were all performed by RH. The author confirms being the sole contributor of this work and has approved it for publication.
